# NVP-BEZ235 (Dactolisib) Has Protective Effects in a Transgenic Mouse Model of Alzheimer’s Disease

**DOI:** 10.3389/fphar.2019.01345

**Published:** 2019-11-13

**Authors:** Paula Maria Quaglio Bellozi, Giovanni Freitas Gomes, Leonardo Rossi de Oliveira, Isabella Guimarães Olmo, Érica Leandro Marciano Vieira, Fabíola Mara Ribeiro, Bernd L. Fiebich, Antônio Carlos Pinheiro de Oliveira

**Affiliations:** ^1^Department of Pharmacology, Universidade Federal de Minas Gerais, Belo Horizonte, Brazil; ^2^Department of Biochemistry and Immunology, Universidade Federal de Minas Gerais, Belo Horizonte, Brazil; ^3^Department of Internal Medicine, Universidade Federal de Minas Gerais, Belo Horizonte, Brazil; ^4^Neuroimmunology and Neurochemistry Research Group, Department of Psychiatry and Psychotherapy, Medical Center—University of Freiburg, Faculty of Medicine, Freiburg im Breisgau, Germany

**Keywords:** Alzheimer’s disease, dactolisib, NVP-BEZ235, neurodegeneration, neuroinflammation, PI3K, mechanistic target of rapamycin

## Abstract

Alzheimer’s disease (AD) is a neurodegenerative disease and the main cause of dementia. Its major symptom is memory loss, which is a result of neuronal cell death, which is accompanied by neuroinflammation. Some studies indicate the overactivation of the phosphatidylinositol 3-kinase (PI3K)/protein kinase B (Akt)/mechanistic target of rapamycin (mTOR) pathway in this disease, being, thus, a potential target for pharmacological treatment. Here, we used a transgenic mouse model of AD that expresses a mutant amyloid-β precursor protein (T41 mice) to investigate the effects of dactolisib (alternative name: NVP-BEZ235, abbreviation BEZ), a dual PI3K/mTOR inhibitor. Ten-months-old T41 animals were treated for 14 days with BEZ or vehicle *via* oral gavage and then submitted to social memory, open field and contextual conditioned fear tests. Hippocampal slices were prepared and Aβ_1-42_ content, NeuN, Iba-1, CD68 and GFAP were evaluated. Tissues were further processed to evaluate cytokines levels through cytometric bead array. The treatment with BEZ (5 mg/kg) reduced social memory impairment in T41 mice. However, BEZ did not have any effect on altered Aβ levels, NeuN, or GFAP staining. The drug reduced the CD68/Iba-1 ratio in CA3 region of hippocampus. Finally, BEZ diminished IL-10 levels in T41 mice. Thus, although its mechanisms are not clear, BEZ protects against memory impairment, reduces microglial activation and reestablishes IL-10 levels, revealing beneficial effects, which should be further investigated for the treatment of AD.

## Introduction

Alzheimer’s disease (AD) is the leading cause of dementia and is characterized as a progressive neurodegenerative disease (Romberg et al., 2012), whose main risk factor is aging (O’neill, 2013). The main sign of AD is memory loss (El Haj and Kessels, 2013), which is directly related to hippocampal and cortical dysfunctions (Wirth et al., 2013).

Neuronal toxicity, which occurs as a consequence of amyloid-β (Aβ) accumulation and tau hyperphosphorylation, results in synaptic loss, neuronal death, and brain atrophy (Lazzari et al., 2015; Kocahan and Dogan, 2017), especially in the entorhinal cortex and hippocampus (Hampel et al., 2002; Holtzman et al., 2011). Synaptic density decrease observed in mesial temporal regions in the early stages of AD correlates with cognitive deficits, since these regions are responsible for the formation and storage of new information (Brouillette, 2014).

Another important phenomenon that occurs in AD is neuroinflammation, which can be mediated by glial cells, especially microglia (Hurley and Tizabi, 2013). Despite evidences that microglia initially removes and degrades Aβ (Krabbe et al., 2013), they lose this ability with the progression of the disease, but they are still capable of producing proinflammatory cytokines (Hickman et al., 2008). Hypertrophic reactive astrocytes are also a hallmark of AD (Frost and Li, 2017), and they are found associated with Aβ in the brain (Chun and Lee, 2018). Alongside with microglia, astrocytes can mediate the clearance of Aβ (Cai et al., 2017; Garwood et al., 2017). The increase of several inflammatory cytokines induce activation of these glial cells, which can contribute to Aβ overgeneration, metabolic misbalance, and problems associated with glutamatergic dysfunction and excitotoxicity (Ourdev et al., 2015; Chen et al., 2016; Cai et al., 2017; Gonzalez-Reyes et al., 2017).

Different pathways contribute to the maintenance and progression of AD. The phosphatidylinositol 3-kinase (PI3K)/protein kinase B (Akt)/mechanistic target of rapamycin (mTOR) signaling pathway regulates cell metabolism, growth, and survival (Kitagishi et al., 2012), being also fundamental for healthy aging. Several studies have shown that in AD early stages, abnormal and continuous activation of PI3K/Akt/mTOR signaling occurs, with increased phosphorylation of mTOR, contributing to disease progression and cognitive decline (Bhaskar et al., 2009; O’neill, 2013). PI3Kγ inhibition and the double inhibition of PI3K and mTOR after Aβ intracerebral injection reduced pathological changes associated with AD (Passos et al., 2010; Bellozi et al., 2016). Importantly, inhibitors of PI3K and mTOR enzymes have been developed in order to elucidate their participation in several diseases and as a possible treatment strategy (Mukherjee et al., 2012). Both enzymes can be inhibited by dactolisib (alternative name: NVP-BEZ235, abbreviation BEZ), a drug that has undergone several clinical trials for the treatment of different tumor types (Maira et al., 2008; Clinicaltrials.Gov, 2015).

Considering that the PI3K/Akt/mTOR pathway is involved in neuroinflammation and neurodegeneration, it is important to establish its roles in AD. Although we have recently demonstrated that the dual inhibition of PI3K and mTOR by BEZ reverses neuropathological changes induced by Aβ (Bellozi et al., 2016), it is important to investigate the effects of the drug in other models, such as transgenic animals, which better resemble the pathological condition (Bilkei-Gorzo, 2014). Therefore, we studied the potential neuroprotective effects of BEZ in a transgenic mouse model that overexpresses APP.

## Material and Methods

### Drugs

The following substances were used in this study: NVP-BEZ235 (BEZ; LC Laboratories, Woburn, EUA), 10% ketamine hydrochloride (Syntec, Brazil), and 2% xylazine hydrochloride (Syntec, Brazil).

### Animals

All procedures were approved by the institutional Ethic Committee on Animal Use (protocol 159/2012) and followed the NIH guide for the care and use of laboratory animals. Experiments were conducted using 10-months-old male transgenic Tg (Thy1-APPSweLon) 41Ema (T41) mice and their wild-type (WT) littermates (Faizi et al., 2012). Male T41 mice were kindly donated by Prof. Tony Wyss-Coray (Glenn Center for aging animal facility, Stanford, USA), and mated with C57Bl/6 female, from Animal Care Facilities of Federal University of Minas Gerais. Animals were kept under controlled room temperature (24°C), under 12 h: 12 h light-dark cycle, with free access to food and water.

### Animals Genotyping

We performed DNA extraction from the tail of the animals followed by polymerase chain reaction (PCR) in the presence of primers specific for the mutant APP and electrophoresis in agarose gel to identify the mutant sequences in the region of 364 base pairs.

### Experimental Protocol

Animals were treated by oral gavage with 5 or 25 mg/kg of BEZ, diluted in 1-metyl 2-pirrolidone 10% in PEG 300, or vehicle, once a day for 14 days. According to the genotype (WT or T41) and the treatment (BEZ or vehicle), the animals were divided into five groups: WT + Vehicle, WT + BEZ 25 mg/kg (WT + BEZ 25), T41 + Vehicle, T41 + BEZ 5 mg/kg (T41 + BEZ 5), and T41 + BEZ 25 mg/kg (T41 + BEZ 25). The doses of BEZ were chosen based on previous published data (Bellozi et al., 2016). Animals were weighed every day before drug administration and the volume of vehicle and drug solution administered was 4 ml/kg of animal weight. Behavioral tests started 30 min after the administration of the drug or vehicle.

### Social Memory Test

Social memory test was performed on the 11^th^ day of treatment, in an acrylic box measuring 60 cm × 40 cm × 23 cm, subdivided in three chambers of equal size, with communication to each other through passages. For habituation, WT + Vehicle (n = 11), WT + BEZ 25 (n = 10), T41 + Vehicle (n = 6), T41 + BEZ 5 (n = 6), and T41 + BEZ 25 (n = 6) animals were introduced for 5 min into the central chamber, with both passages closed. To perform the test, two juveniles C57Bl/6 animals, obtained from a different source of the test animals, were used. One min after the habituation phase, the first juvenile was introduced into one of the lateral chambers, inside a compartment. The passages were opened for 10 min. Then, the test animal was driven to the central compartment, the passages were closed, and a new C57Bl/6 juvenile animal was introduced into the other side chamber, inside another compartment. One min after the second phase, the passages were opened and the interaction of the test animal with both juveniles was evaluated for further 10 min. Each trial (the three phases of the test) was performed for each animal before starting the next. The percentage of time spent in the compartment with the new juvenile animals was calculated using the formula: 100*[time exploring compartment with new juvenile animal/(time exploring compartment with old animal + time exploring compartment with new juvenile animal)] (Nadler et al., 2004; Kobayashi and Chen, 2005). Tests were recorded and videos were analyzed using ANY-maze software version 4.99.

### Open Field Test

Open field test was performed on the 12^th^ day of treatment. Animals were introduced in the center of a 30cm diameter open field during 10 min, and the total traveled distance was assessed. Experimental groups and sample sizes were the same used for social memory test.

### Contextual Conditioned Fear Test

On the 13^th^ day of treatment, animals were habituated for 3 min in a conditioning fear chamber containing a grid bottom, dimensions 23 cm × 20 cm × 21 cm. After that, a cycle of 3 shocks of 600 mA for 2 s was started, with intervals of 30, 60, and 40 s, respectively. After the last shock, animals were left into the chamber for 1 min more. On the following day, they were reintroduced into the chamber, and the freezing time was measured during 5 min (Roy et al., 2016). Experimental groups and sample sizes were the same used for social memory test.

### Intracardiac Perfusion, Brain Slice Preparation, and Tissue Dissection

On the last day of treatment, 1 hour after drug administration, a subgroup of animals [WT + Vehicle (n = 7), T41 + Vehicle (n = 6) and T41+BEZ 5 (n = 6)] was intraperitoneally anesthetized with ketamine (80 mg/kg) and xylazine (8 mg/kg) and perfused with PBS. Then, animals were decapitated, brains were removed, stored in PFA 4% overnight, and subsequently moved to a 30% sucrose solution until complete saturation. Brains were frozen, stored at −80°C (Gage et al., 2012), and posteriorly sliced into 30-µm-thick sections at −20°C with the aid of a cryostat.

Another subgroup of animals [WT + Vehicle (n = 11), T41 + Vehicle (n = 6) and T41 + BEZ 5 (n = 5)] had their hippocampus carefully dissected 1 h after drug administration, and stored at −80°C, until the day of analysis.

### Histological Analysis

Free-floating slices from perfused animals were incubated with citrate buffer at 70°C for 1 h for antigen retrieval, followed by blocking solution [BSA (4%), Triton X (0.5%) in TBS] for 1 h. Then, the primary antibodies rabbit anti-Fox3/NeuN (1:800; EnCor, USA); rabbit anti-Aβ _1-42_ (1:1,600; 1-42 specific; D9A3A, Cell Signaling, USA), mouse anti-GFAP (1:800; Cell Signaling, USA), rabbit anti-Iba-1 (1:500; Wako, Japan), or rat anti-CD68 (1:500; Bio-Rad, USA) were added and incubated overnight. On the next day, the secondary antibodies donkey antirabbit (1:1,000; Alexa Fluor 594, Invitrogen, USA), goat antimouse (1:1,000, Alexa Fluor 488, Life Technologies, USA)or goat antirat (1:1,000, Alexa Fluor 488, Life Technologies, USA) were added for 1 h. Slices were mounted in gelatinized slides and coverslipped with Fluoromount media (Sigma-Aldrich, USA).

Immunostaining was analyzed under a Zeiss fluorescence microscope in 20X/0.4NA magnification lens. In order to obtain a broad and representative perspective of the whole region that was being evaluated, separated slices ranging from −2.06 to −2.54 mm relative to bregma were used. In order to make a proper comparison, equivalent regions containing similar portions were chosen for all the groups. Three slices per animal were used. One picture per region (CA1, CA3, or DG) of each of the three slices were taken. Each picture contained the oriens, pyramidal, and lacunous-molecular layers in the case of CA1 and CA3 regions. In the case of DG, the molecular, granular and polymorphic layers were included in the images and all of them were analyzed together. Each picture had dimension of 710 µm × 530 µm (1388 × 1040 pixels) and resolution of 1.96 pixels/µm. The boundaries of the CA1, CA3, and DG layers of hippocampus were determined by anatomical delimitation as previously established (George Paxinos, 2001). Photomicrographs of stained fluorescence were quantified with the aid of ImageJ software (NIH), and the whole picture was used for quantification. To perform immunostaining analysis, images were converted to 8-bits type, then the threshold method with default algorithm was applied, followed by the percentage of area occupied, mean intensity or integrated density quantification by the analyze particle method, with size (pixels^2) of 0-infinity range and circularity of 0.0–1.0 range. For GFAP, NeuN, Iba-1, and CD68 staining, threshold level was selected as automatically provided by the software. For Aβ_1-42_ staining, it was established a minimum size of 20 pixels (9 µm) with the analyze particle tool in order to avoid the detection of unspecific objects. The integrated density (IntDen) reflects the product between the area and the mean gray value and it was used in addition to mean intensity. In order to evaluate the percentage of Iba-1-stained area counterstained by CD68, CD68/Iba-1 ratio was obtained by the ratio between CD68 and Iba-1 stained areas. Finally, the number of Iba-1^+^ cell clusters was counted using 10X (0.25 of numerical aperture) magnification photomicrographs.

### Cytokine Analysis

Hippocampi from dissected animals were homogenized in 200 µl of a buffer containing protease inhibitors (0.4 M NaCl; 0.05% Tween 20; 0.5% BSA; 0.1 mM phenylmethylsulfonyl fluoride; 0.1 mM benzethonium chloride; 10 mM EDTA; 20 IU aprotinin in PBS). Total proteins were measured by Bradford method (Bradford, 1976) and analyzed by cytometric bead array (CBA) with the Th1/Th2 kit (BD, USA) to detect IL-2, IL-4, IL-5 IL-6, IFN-γ, TNF-α, IL-10, and IL-17A. All the procedures followed manufacturer’s instructions.

### Statistical Analysis

Statistical analysis was performed using the statistical software graphpad prism 6.0 and statistica 7.0. Quartile extreme test for identification of outlier values was applied on the results, and extreme values were removed before analysis with the aid of the interquartile range method. body weight data were analyzed by two-way Analysis of Variance (ANOVA), followed by Newman-Keuls Posttest. Behavioral, biochemical, and histological data were analyzed by one-way ANOVA followed by Newman-Keuls post-test. the data were presented as mean ± standard error of the mean (SEM). The level of significance was set At P < 0.05.

## Results

### BEZ Does Not Alter Body Weight

**Figure 1:**
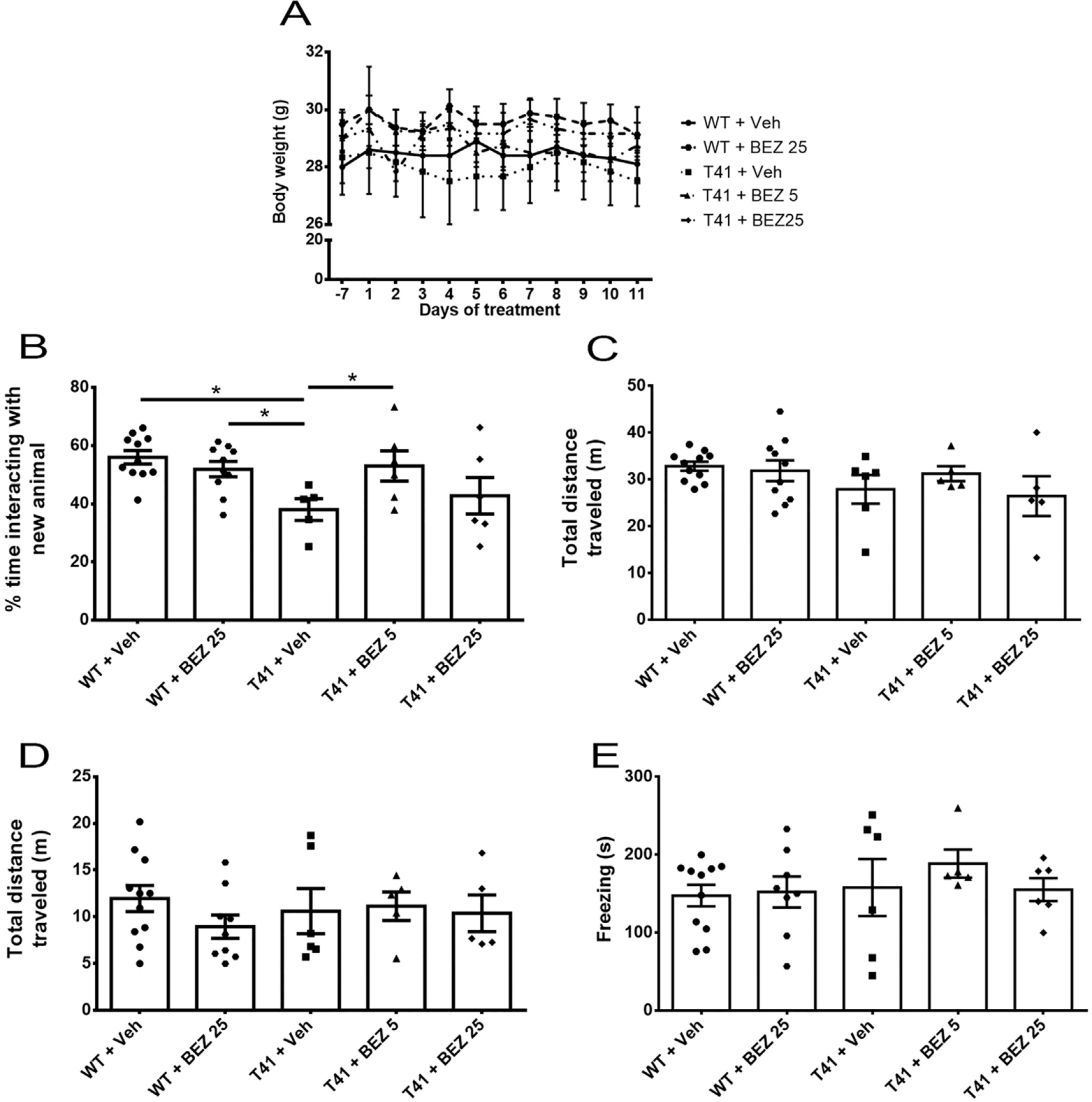
Effects of BEZ treatment on mice weight, memory, and locomotor activity. **(A)** Lines represent the quantification of BEZ treatment effect on animal’s weight; **(B)** Bar graphs represent the quantification of BEZ treatment effect on animal’s social memory; **(C)** Total traveled distance during the social memory test; **(D)** Total traveled distance in open field test; and **(E)** freezing time in the contextual conditioned fear test. WT + Vehicle (n = 11), WT + BEZ 25 (n = 10), T41 + vehicle (n = 6), T41 + BEZ 5 (n = 6) and T41 + BEZ 25 (n = 6). *p < 0.05 (ANOVA followed by Newman-Keuls test).

We first tested whether BEZ would alter body weight, in the same groups of animals submitted to behavioral tests. We did not observe differences between experimental groups (F(4,12) = 0.804, p = 0.545) and there was no interaction between variables (F(44,132) = 1.290, p = 0.137). However, there was effect of the variable time, with reduction of the mean body weight in all experimental groups (F(11,132) = 2.123; p = 0.022; **Figure 1A**).

### BEZ Rescues Social Memory in T41 Mice

Memory loss is the main sign of AD. Therefore, we aimed to evaluate T41 animal’s cognition in the social memory test. T41 mice explored for less time the new juveniles compared to WT + Vehicle and WT + BEZ 25 groups. Importantly, BEZ 5 mg/kg significantly reversed this memory impairment in T41 mice [F(4,33) = 3.596; p = 0.014; **Figure 1B**]. We did not observe any difference in total distance traveled in the apparatus between groups [F(4,32) = 1.308; ns; **Figure 1C**]. Mean ± SEM of the total time of exploration of each compartment in 1^st^ and 2^nd^ sessions and the total distance traveled in the 1^st^ session can be found in **Supplementary Figure 1**.

### BEZ Does Not Change Locomotor Activity in T41 Mice

Memory loss may be accompanied by locomotor changes. Thus, we also evaluated animal’s locomotor activity in an open field. Treated and nontreated T41 mice did not exhibited any locomotor changes when compared with WT treated or nontreated groups [F(4,31) = 0.577; ns; **Figure 1D**].

### T41 Mice Do Not Reveal Altered Behavior in Contextual Conditioned Fear Test

Contextual conditioned fear test was used to assess memory through another behavioral paradigm. However, T41 mice did not present memory impairment in this task and both BEZ 5 and 25 mg/kg did not change the behavior [F(4,31) = 0.494; ns; **Figure 1E**].

### BEZ Does Not Change the Increase in Aβ_1-42_ Plaque Load in the Hippocampus of T41 Mice

**Figure 2:**
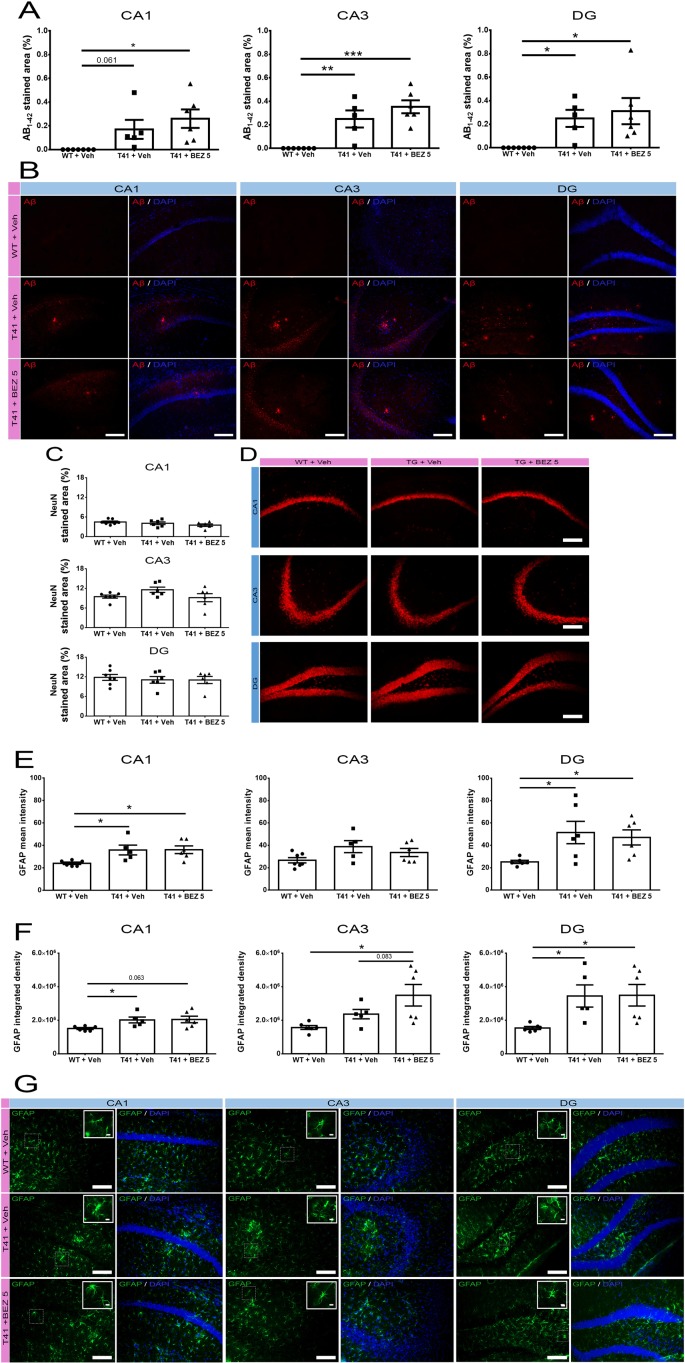
Effects of BEZ treatment on Aβ, NeuN, and GFAP immunostaining. **(A**, **B)** Bar graphs represent the quantification of BEZ treatment effect on Aβ; **(C**, **D)** NeuN; **(E**–**G)** GFAP immunostaining. WT + Vehicle (n = 7), T41 + vehicle (n = 6), and T41 + BEZ 5 (n = 6). Representative images of Aβ **(B)**, NeuN **(D)**, and GFAP **(G)** immunostaining in CA1, CA3, and DG of animals. Inset images are a magnified portion to show detail (40× objective). *p < 0.05, **p < 0.01, and ***p < 0.001 (ANOVA followed by Newman-Keuls test). Scale bar for 20× objective = 125 µm. Scale bar for 40× objective = 10 µm.

Considering that the higher dose of BEZ did not alter the behavior tasks, we further evaluated the effect of the lower dose in different parameters associated with AD. Since we observed that BEZ rescued social memory in T41 mice, we investigated whether this effect was related to changes in Aβ plaque load. Aβ_1-42_ was increased in CA1 (F(2,15) = 5.496; p = 0.0162), in CA3 (F(2,15) = 16.47; p = 0.0002) and in the DG (F(2,15) = 5.606; p = 0.0152) of the hippocampus of T41 mice treated with vehicle. BEZ treatment did not reverse the increased Aβ_1-42_ load observed in T41 mice (**Figures 2A, B**).

### BEZ Does Not Alter the Immunostaining of NeuN in the Hippocampus of T41 Mice

In order to further investigate the possible neuroprotective effect of BEZ, we evaluated its effect in NeuN immunostaining. There was no alteration in NeuN stained area in CA1 (F(2,16) = 1.798; ns), CA3 (F(2,16) = 2.252; ns), and DG (F(2,16) = 0.2116; ns) regions of the hippocampus, when comparing WT and T41 nontreated or treated mice (**Figures 2C, D**).

### T41 Mice Present Astrocytosis in the Hippocampus, Which Is Not Altered by BEZ

Astrocytosis is a common event observed in AD that may also contribute to the progression of the disease. Therefore, we evaluated whether T41 mice presented changes in GFAP intensity and whether BEZ would alter this scenario. There was an increase in the GFAP mean intensity (F(2,14) = 5.132; p = 0.021) and GFAP IntDen (F(2,14) = 4.077; p = 0.040) in CA1 in nontreated T41 mice, as compared with their WT littermates. In CA3 region, we observed only a trend towards an increase in GFAP mean intensity (F(2,15) = 2.757; ns) but an increase in GFAP IntDen (F(2,14) = 5.468; p = 0.017) in nontreated T41 group, as compared to nontreated WT group. In the DG, both GFAP mean intensity (F(2,15) = 4.044; p = 0.039) and IntDen (F(2,14) = 4.762; p = 0.026) were significantly increased in nontreated T41 mice, in comparison with WT. We also noticed that the treatment did not reverse the increase in neither GFAP mean intensity nor GFAP IntDen found in nontreated T41 mice. In addition, we observed an increase in GFAP IntDen in CA3 of treated T41 mice, as compared with nontreated WT mice (**Figures 2E–G**).

### BEZ Partially Reduces the Increased Microglial Activation in the Hippocampus of T41 Mice

**Figure 3:**
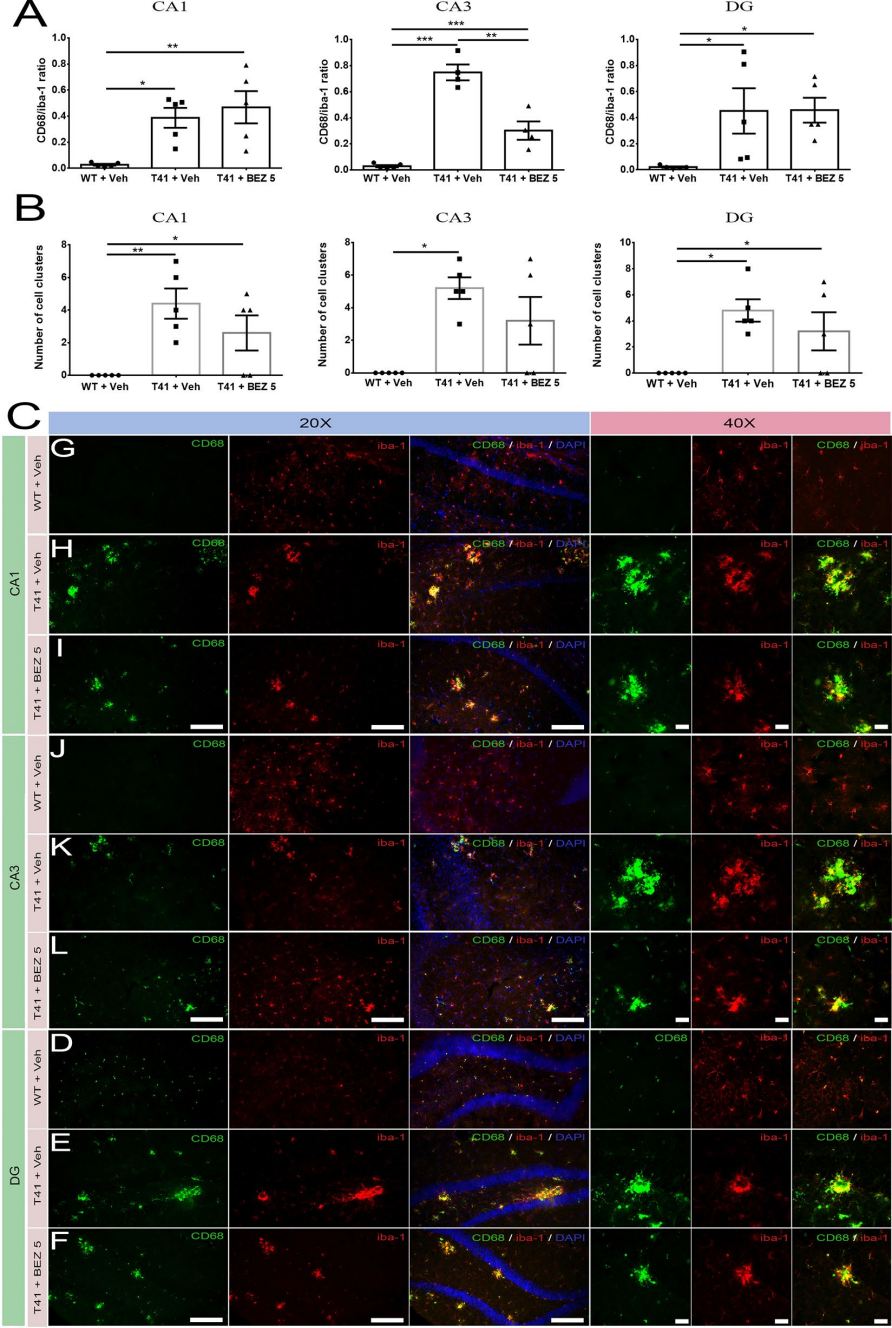
Effects of BEZ treatment on CD68 and Iba-1 immunostaining ratio. Bar graphs represent the quantification of BEZ treatment effect on ratio between CD68 and Iba-1 immunostaining **(A)** or the number of Iba-1^+^ cell clusters **(B)** in CA1, CA3, and DG. Representative images are shown in 20× and 40× magnitude **(C)**. For CD68 and Iba-1 immunofluorescence, it was used WT + Vehicle (n = 5), T41 + vehicle (n = 5), and T41 + BEZ 5 (n = 5). *p < 0.05, **p < 0.01, and ***p < 0.001 (ANOVA followed by Newman-Keuls test). Scale bar for 20× objective = 125 µm. Scale bar for 40× objective = 10 µm.

Microglia activation may be a result of neurodegeneration, as well as it could also contribute to the progression of the disease. Since CD68 has been suggested as a lysosomal marker highly expressed in activated macrophages/microglia, and Iba-1 is a marker of microglia, we evaluated whether BEZ would also reduce the ratio between CD68 and Iba-1 immunostaining. Importantly, we did not notice CD68 staining out of Iba-1^+^ cells. There was an increase in CD68/Iba-1 ratio in CA1 (F(2,12) = 7.796; p = 0.0068), CA3 (F(2,10) = 55.14; p < 0.0001), and DG (F(2,12) = 4.775; p = 0.0298) layers in both T41 nontreated and treated mice. Importantly, BEZ reduced the increase in CD68/Iba-1 ratio in CA3 layer of T41 mice. In addition to the CD68/Iba-1 ratio, we also counted the number of Iba-1^+^ cell clusters in each layer. We considered as a cluster every Iba-1^+^ cell agglomerate, which probably occurs around the plaques. The number of cell clusters was increased in CA1 (F(2,12) = 7.267; p = 0.0086) and in DG (F(2,12) = 6.222; p = 0.014) layers in both T41 nontreated and T41 treated mice in comparison with the WT littermates. In CA3 layer, there was an increase in the number of cell clusters in T41 nontreated mice, as compared to WT nontreated mice (F(2,12) = 8.00; p = 0.0062; p < 0.01). However, there was no post-test difference between T41 treated mice and WT nontreated mice or between T41 treated and T41 nontreated mice (**Figure 3**).

### BEZ Reduces Hippocampal IL-10 in T41 Mice, But No Other Cytokines Are Changed

**Figure 4:**
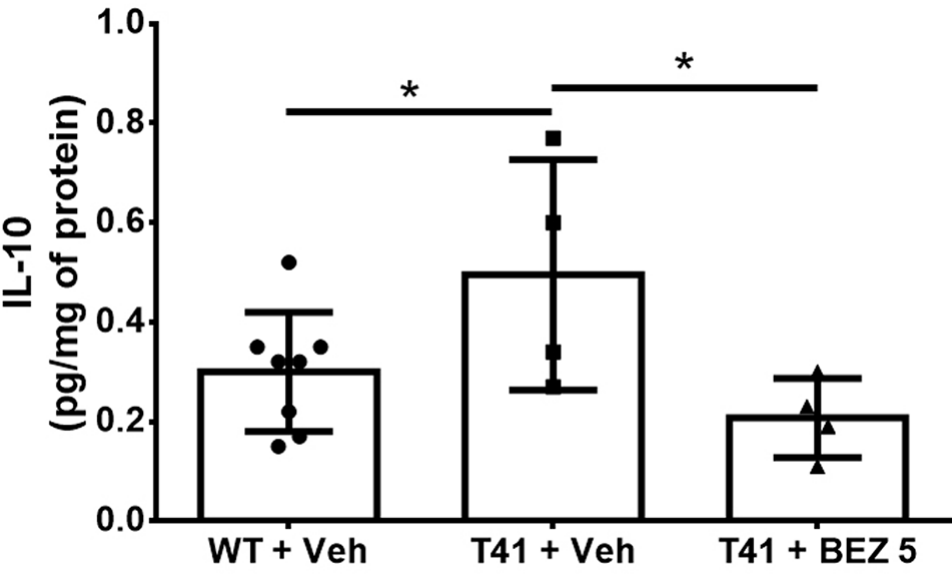
Effects of BEZ treatment on mice hippocampal IL-10 levels. Bar graph represent the quantification of the effect of BEZ treatment on IL-10 levels in the hippocampus. WT + Vehicle (n = 11), T41 + vehicle (n = 6), and T41 + BEZ 5 (n = 5). *p < 0.05 (ANOVA followed by Newman-Keuls test).

Finally, we evaluated the effects of BEZ on cytokines levels. Among the cytokines evaluated (**Supplementary Table 1**), only IL-10 was found to be increased in T41 mice, which was reversed by treatment with BEZ [F(2,13) = 4.079; p < 0.05; **Figure 4**].

## Discussion

In the present study, we demonstrated that BEZ reversed memory impairment and levels of hippocampal IL-10 in 10-months-old T41 mice. Besides, it reduced CD68/Iba-1 immunostaining ratio in CA3 region of these transgenic animals.

Increased Akt activation and mTOR phosphorylation have been reported in brains of AD patients, which is associated with a disrupted clearance of Aβ and tau, synaptic loss, and cognitive decline (Heras-Sandoval et al., 2014). Thus, the adequate control of PI3K/Akt/mTOR pathway activation might have a potential to ameliorate AD associated features. The dual inhibition of PI3K and mTOR with BEZ 5mg/kg reduced memory loss in T41 mice, without, however, changing the locomotor parameter. The higher BEZ dose did not induce significant memory improvement in this mice strain aged 10 months old. The ineffectiveness of this dose should be evaluated in future studies. It was previously demonstrated that the inhibition of PI3K with LX2343 ameliorates memory loss in APP/PS1 transgenic mouse (Guo et al., 2016). Moreover, the inhibition of mTOR with rapamycin rescues memory impairment (Spilman et al., 2010), since it regulates autophagy leading to a reduction of APP levels, processing and metabolites production, and oxidative stress (Tramutola et al., 2018). Although further studies are necessary, these mechanisms may be related to the BEZ effects in the present study.

PI3K and mTOR inhibition have neuroprotective effects in different mice models of neurodegeneration (Malagelada et al., 2010; Bellozi et al., 2016; Saliba et al., 2017). In animal models of AD, administration of rapamycin reduces the accumulation of Aβ, leading to the reduction of synaptic neurotransmission dysfunction (Spilman et al., 2010; Singh et al., 2017). Furthermore, dual inhibition of PI3K and mTOR with BEZ reduces memory impairment induced by intrahippocampal injection of Aβ, which is associated with reduced neurodegeneration and reduced microglial activation (Bellozi et al., 2016). In the present investigation, 14 days of treatment with BEZ did not evidence changes in Aβ_1-42_ immunostaining, when compared with T41 animals, suggesting that the cognitive improvement mediated by BEZ is related to another mechanism, instead of the modulation of Aβ processing. Since it was previously demonstrated that BEZ reduces Aβ-induced neurodegeneration (Bellozi et al., 2016), we evaluated NeuN immunostaining in T41 mice to assess its potential protective effect. We evaluated CA1, CA3, and DG, since these regions are involved in memory and the neuronal circuits involving them may be affected in AD (Brouillette, 2014; Moorthi et al., 2015). Nevertheless, we did not observe changes in the density of neurons, as indicated by the percentage of stained area with anti-NeuN antibody, nor did BEZ alter this parameter. However, further estimation of the total NeuN^+^ cells could contribute to the understanding of the possible neuroprotective effect of the tested drug.

Aβ accumulation also leads to neuroinflammation in AD, which is primarily mediated by microglia and astrocytes (Giovannini et al., 2002; Hurley and Tizabi, 2013; Janota et al., 2015). Herein, we observed astrocytosis in hippocampi of T41 animals. It is important to note that GFAP is also expressed by radial glia-like stem cells in the DG. However, BEZ did not induce significant effects in GFAP expressing cells in T41 mice. In addition, we also evaluated the microglia profile. It is known that microglia response can significantly contribute to the chronic neuroinflammation, which has strong relationship with the progression of AD (Li et al., 2018; Nordengen et al., 2019). BEZ reduced the increased CD68/Iba-1 ratio in CA3 region of transgenic animals, suggesting an interference in microglial activation, since CD68 has been suggested as a lysosomal marker highly expressed in activated macrophages/microglia (Graeber and Streit, 2010; Lehmann et al., 2016; Navarro et al., 2018). Moreover, despite treatment with BEZ did not significantly reduce the number of Iba-1 cell clusters in CA3 of T41 mice, there were no differences between the treated T41 group and WT animals. Despite the decrease in the CD68/Iba-1 ratio induced by BEZ was restrained to CA3 region of T41 mice, it is worth highlighting that the treatment was performed for only 14 days. Thus, although it is possible to speculate that the drug may interfere with microglia activity and neuroinflammatory process, this pharmacological effect should be further investigated. It is equally relevant to point out the limitations of bidimensional (2D) evaluations, such as the lack of volume data and the reduction of information regarding objects in the three-dimensional (3D) structure (Boyce et al., 2010; Fujisawa et al., 2015), which could be further used to corroborate the present histological findings.

Altered cytokine gene expression and protein levels are also associated with the neuroinflammatory process of AD (Strauss et al., 1992; Janelsins et al., 2008; Morimoto et al., 2011), having protective or nonprotective roles (Su et al., 2016; Zheng et al., 2016). Interestingly, we observed elevated levels of IL-10 in the hippocampus of T41 mice, which was reversed by BEZ. Our finding corroborates previous reports of elevated IL-10 levels in patients (Asselineau et al., 2015) and AD mouse models (Jin et al., 2008). However, in our previous study BEZ increased IL-10 hippocampal levels (Bellozi et al., 2016), a difference that may be related to the age of the animal and stimuli used. Although it is assumed that IL-10 has an antiinflammatory role, its function in AD is still controversial, since it could be either protective or deleterious (Bellozi et al., 2016; Lobo-Silva et al., 2016). We did not find alterations in the other evaluated cytokines in the hippocampus of neither nontreated nor treated T41 animals, as compared to WT mice, which might probably be due to the age.

The effects observed in the present study were not due to body weight change, since the alteration of this parameter along the time was less than 5% and there was no difference between the groups of animals. When comparing the present study results with the model using a hippocampal injection of Aβ, we must consider that the transgenic model better mimics AD, leading to Aβ accumulation in the whole brain (Rockenstein et al., 2001). Herein, a 14-day treatment in a transgenic mouse model expressing a mutant APP was already enough to detect an important cognitive improvement and some benefits related to neuroinflammation, albeit it was not able to change other pathological features. Thus, other mouse models, longer treatment duration or an earlier intervention should be considered to fully understand the potential of BEZ in the treatment of AD.

## Data Availability Statement

The datasets generated for this study are available on request to the corresponding author.

## Ethics Statement

All procedures were approved by the institutional Ethic Committee on Animal Experimentation from Federal University of Minas Gerais (CEUA/UFMG) under the protocol number 159/2012. Procedures are in agreement with the Ethical Principles in Animal Experimentation, adopted by CEUA/UFMG, and followed the National Institutes of Health guide for the care and use of Laboratory animals.

## Author Contributions

PB, BF, and AO designed the study. IO did the animal genotyping. PB was responsible for animal breeding, performing the treatments, behavioral tasks, removal of tissues, intracardiac perfusion, and slices and tissues preparation. LO helped in behavioral tasks and intracardiac perfusion. GG did histological staining. Cytokines dosages were done by ÉV and PB. Results were analyzed by PB and GG. Article was written by PB, GG, BF, and AO. Authors PB, GG, FR, BF, and AO revised the data and discussed and corrected the manuscript.

## Funding

Fundação de Amparo à Pesquisa do Estado de Minas Gerais (FAPEMIG; process numbers CBB-APQ-02044-15 and APQ-02559-17), Conselho Nacional de Desenvolvimento Científico e Tecnológico (CNPq - 424588/2016-1) and Coordenação de Aperfeiçoamento de Pessoal de Nível Superior (CAPES). The project was in part supported by the AIF project GmbH (BMWi) (AGEsense).

## Conflict of Interest

The authors declare that the research was conducted in the absence of any commercial or financial relationships that could be construed as a potential conflict of interest.

## Abbreviations

AD, Alzheimer’s disease; Aβ, amyloid-β; APP, amyloid precursor protein; BEZ, NVP-BEZ235; BSA, bovine serum albumin; CBA, cytometric bead array; CA, cornus ammonis; DG, dentate gyrus; i.p., intraperitoneal; PBS, phosphate buffered saline; SEM, standard error of the mean; TBS, tris buffered saline; TBST, TBS-Tween-20; T41, Tg(Thy1-APPSweLon)41Ema.
