# Supplementary material for: NVP-BEZ235 (Dactolisib) Has Protective Effects in a Transgenic Mouse Model of Alzheimer’s Disease
Source: Front Pharmacol. 2019 Nov 13;10:1345. doi: 10.3389/fphar.2019.01345 (PMC6864823; doi:10.3389/fphar.2019.01345)
Supplement: Supplementary file 1 [file Table_1.pdf]

Cytokine	F	p
IL-2	F(2,18)=0.834	ns
IL-4	F(2,18)=0.568	ns
IL-5	F(2,18)=1.063	ns
IL-6	F(2,14)=2.689	ns
IFN-γ	F(2,18)=0.411	ns
TNF-α	F(2,18)=0.733	ns
IL-17A	F(2,14)=0.395	ns

Supplementary Table 1. BEZ effect on cytokines. Statistical analysis of the concentration of cytokines in the hippocampus of animals from groups WT + vehicle, T41 + vehicle and T41 + BEZ (ANOVA followed by the Newman-Keuls post-test).
