# Supplementary figures and images for: NVP-BEZ235 (Dactolisib) Has Protective Effects in a Transgenic Mouse Model of Alzheimer’s Disease
Source: Front Pharmacol. 2019 Nov 13;10:1345. doi: 10.3389/fphar.2019.01345 (PMC6864823; doi:10.3389/fphar.2019.01345)

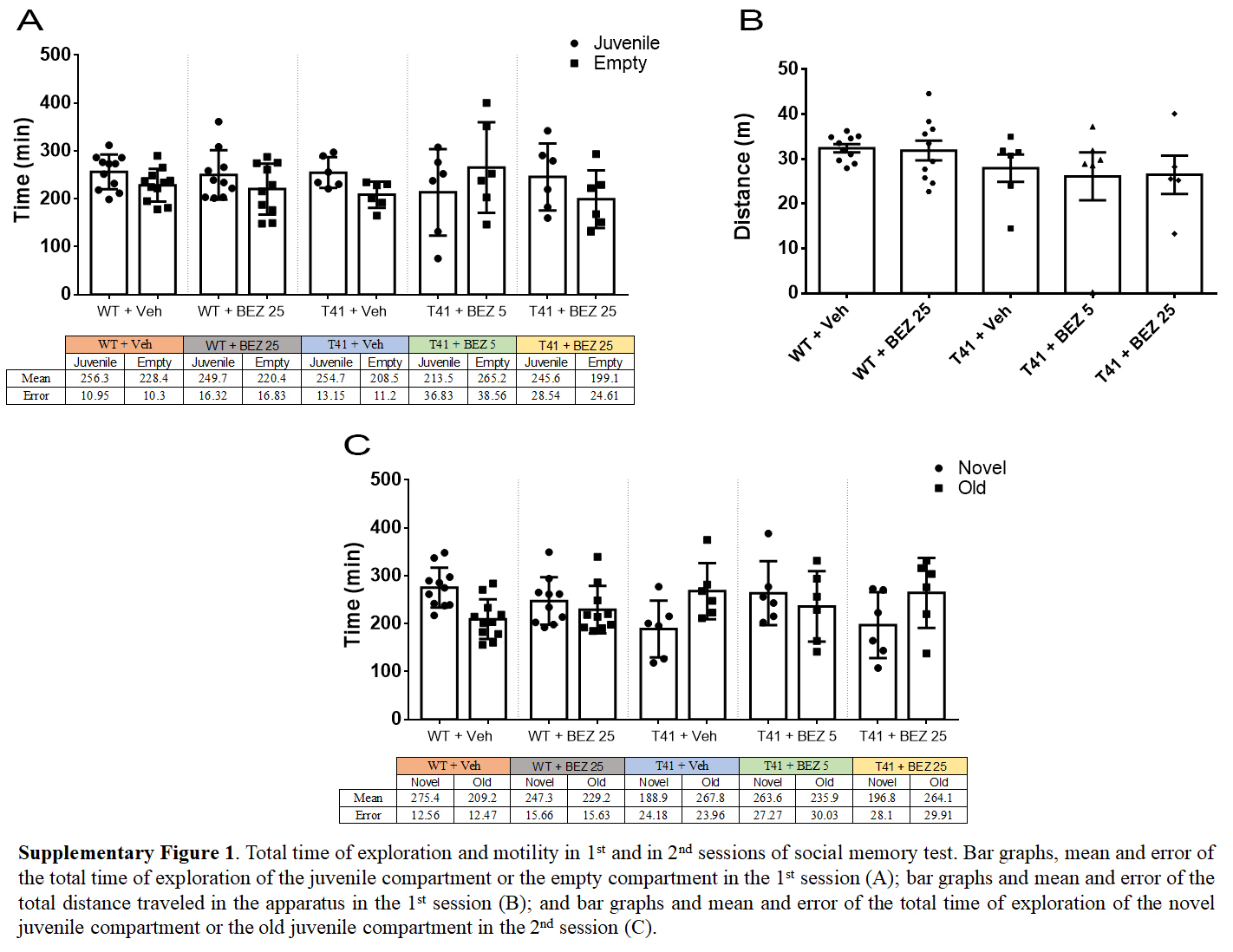

Supplement: Supplementary file 2 [file Image_1.jpg]
